# The Use of Point-of-Care Ultrasound in the Diagnosis and Percutaneous Aspiration of Liver Abscess in a Resource-Limited Country: A Case Report

**DOI:** 10.7759/cureus.63905

**Published:** 2024-07-05

**Authors:** Sheikh Omar Bittaye, Syadiba Tamba, Mariam Jaw, Sidat Joof, Ian Pelletier, Jessica Pelletier

**Affiliations:** 1 Internal Medicine and Gastroenterology, Edward Francis Small Teaching Hospital, Banjul, GMB; 2 Internal Medicine, School of Medicine and Allied Health Sciences, University of the Gambia, Banjul, GMB; 3 Internal Medicine and Hepatology, Edward Francis Small Teaching Hospital, Banjul, GMB; 4 Internal Medicine, Edward Francis Small Teaching Hospital, Banjul, GMB; 5 Internal Medicine, Missouri Highlands Healthcare, Ellington, USA; 6 Emergency Medicine, Washington University School of Medicine in St. Louis, St. Louis, USA

**Keywords:** clinical case report, gastroenterology, low-resource setting, point-of-care-ultrasound (pocus), liver abscess

## Abstract

Diagnosis and management of liver abscesses in low- and middle-income countries (LMICs) is difficult due to limited diagnostic imaging availability. Limited data is available describing the use of point-of-care ultrasound (POCUS) in the diagnosis and percutaneous aspiration of liver abscesses in resource-limited countries. We describe a 21-year-old female who was diagnosed with a liver abscess. The diagnosis of liver abscess was made via POCUS, and the patient was successfully managed with empiric antimicrobials and repeated POCUS-assisted percutaneous needle aspiration. In resource-limited settings, adequate training of personnel and availability of POCUS may help in early diagnosis and treatment of liver abscess - thus helping to reduce its related morbidity and mortality - while also aiding in resource conservation.

## Introduction

Liver abscesses are an important cause of hospitalization in low- and middle-income countries (LMICs). The etiology is typically amoebic or pyogenic [1]. Amoebic liver abscess is primarily caused by *Entamoeba histolytica*, a protozoan. Though *E. histolytica* is globally distributed, infection rates are higher in LMIC settings due to poor living conditions and contamination of drinking water [1]. Amoeba are transmitted via the fecal-oral route, and the incubation period varies widely. An amoebic liver abscess can form when *E. histolytica* trophozoites travel to the liver via the portal vein. Microabscesses form, which then coalesce to form one large abscess [2]. This disease process typically affects males 20-40 years of age. Liver function tests and eosinophil counts in patients with amoebic liver abscesses may be within normal limits, and neutrophilia may be present. Amoebic liver abscesses may rupture, become superinfected, or lead to the seeding of other organs via hematogenous spread [3].

Pyogenic liver abscesses are bacterial in origin with *Escherichia coli*, *Klebsiella *species, *Streptococcus* species, and *Staphylococcus *species being the most common organisms. Anaerobic infections are usually polymicrobial. The most common source of infection is bowel leakage, but other causes include peritonitis and biliary transmission. *Klebsiella *infections are associated with colorectal cancer, and sources of hematogenous spread such as endocarditis should be investigated in cases of *Streptococcus *and *Staphylococcal *pyogenic liver abscess [4].

Ultrasound (US) is a diagnostic tool that is heavily utilized in all medical settings to aid in the management of infectious diseases due to its overall low cost and point-of-care US (POCUS) protocols are proliferating. However, the use of POCUS in the management of tropical infectious diseases has received less attention, despite the incredible potential for POCUS to improve care in resource-limited settings [5]. Limited data is available describing the use of POCUS in the diagnosis and percutaneous aspiration of liver abscess by the primary medical team (i.e. non-radiologists) in LMICs based on a literature review of PubMed, Embase, and Google Scholar; only one prior case report was identified [6]. This case report therefore highlights the critical role of POCUS for the early diagnosis and aspiration of this potentially life-threatening condition, as well as the potential that this may be conducted safely outside of the radiology suite.

## Case presentation

A 21-year-old previously healthy female was referred from a district hospital to the national referral hospital with a suspected clinical diagnosis of gastric tumor. The patient presented with a four-day history of abdominal pain and distension associated with diarrhea, fever, weight loss, loss of appetite, and vomiting. On examination, the patient was acutely ill-appearing, pale, and icteric, with no pedal edema. Vital signs showed blood pressure of 124/82 mmHg, pulse rate of 121 beats/minute, respiratory rate of 22 cycles/minute, and temperature of 37.5°C. Her weight at the presentation was also 44.5 kg. Her abdomen was distended with smooth, tender hepatomegaly noted.

**Figure 1:**
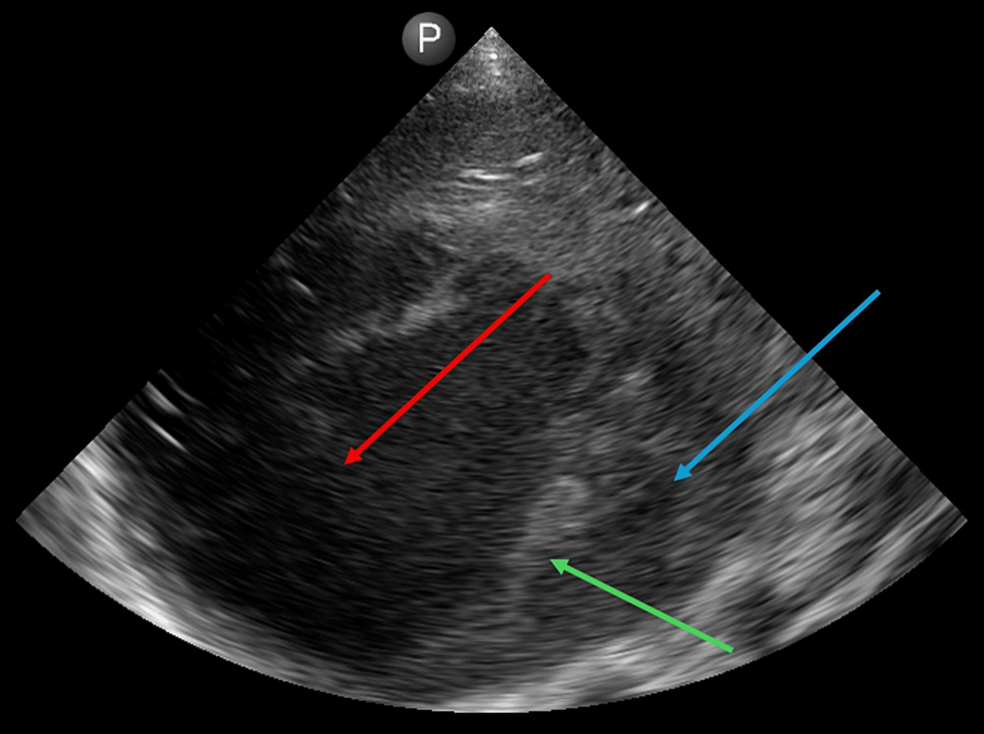
POCUS image of a hypoechoic liver mass with heterogenous echotexture and a hyperechoic rim in the peripheral area of the liver at the time of first drainage. POCUS: point-of-care ultrasound. The blue arrow indicates the liver, the red arrow indicates the abscess, and the green arrow indicates the hyperechoic rim.

Diagnostic workup revealed a negative hepatitis C virus antibody, hepatitis B surface antigen, and human immunodeficiency virus tests. Her full blood count results showed normocytic anemia with a hemoglobin level of 6.7 g/dL with neutrophilic leukocytosis (white blood cell count of 24 x 10^9^/L, 85% neutrophils). A random blood sugar was 9.2 mmol/L. Stool microscopy was negative for ova and parasites, and there was no stool culture growth. Liver function tests revealed an alanine transferase level of 148 U/L, aspartate transferase of 316 U/L, alkaline phosphatase of 201 U/L, and albumin of 30.5 g/L. Her erythrocyte sedimentation rate (ESR) was 130 mm/hr. A formal abdominal US by a radiology technician showed a complex mass, measuring 9.8 x 4.8 cm in the right lobe of the liver suggestive of a liver tumor. As the clinical history and laboratory investigations did not support the diagnosis of a liver tumor, POCUS was performed at the bedside (Figure 1), and a hypoechoic mass measuring about 10 x 5 cm in the right lobe of the liver was found with some hyperechoic areas in the periphery of the lesion, features suggestive of liver abscess. A chest X-ray also showed a right pleural effusion.

**Figure 2:**
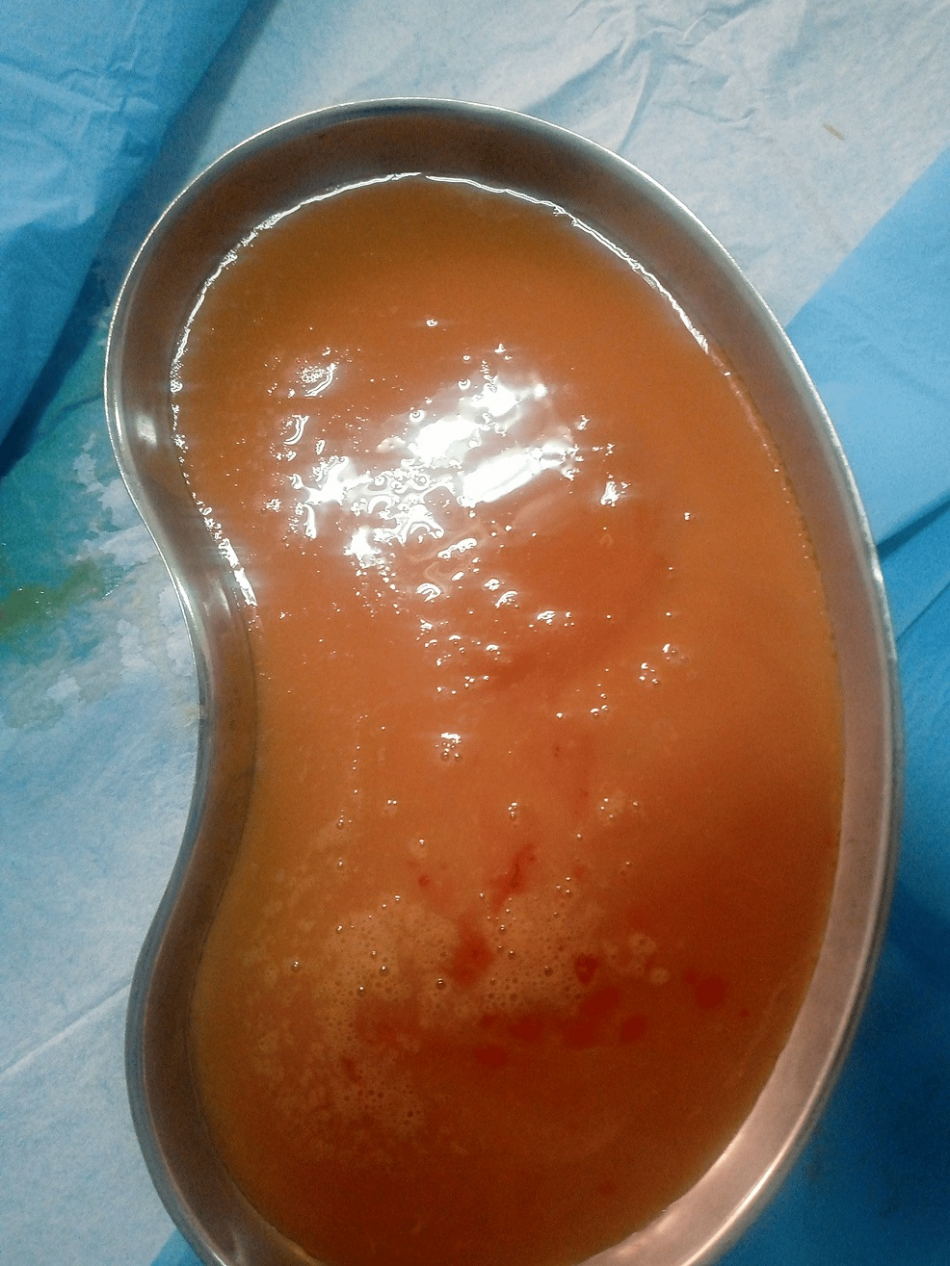
During admission, 1.3 liters of thick, odorless, paste-like pus was aspirated from the abscess over two sessions.

The patient was admitted and started on intravenous (IV) ciprofloxacin 500 mg twice daily for one week and IV metronidazole 800 mg three times daily for two weeks (which is standard dosing in the Gambia). Percutaneous POCUS-assisted needle aspiration of the liver abscess was performed by gastroenterology using the sterile technique on days 9 and 12 of admission with the removal of 655 mL and 485 mL of pus, respectively. This procedure was conducted by the primary medical team at the bedside, eliminating the need for the patient to be transferred to the radiology suite and involve another consultant. The pus was an odorless, brownish paste, prototypical for an amoebic liver abscess (Figure 2). There was no bacterial growth on culture and confirmatory antibody testing for *E. histolytica* is unavailable in the Gambia; therefore, the patient was treated empirically for amoebic liver abscess. The patient was discharged after 15 days of admission and prescribed a two-month course of oral metronidazole 800 mg three times daily.

**Figure 3:**
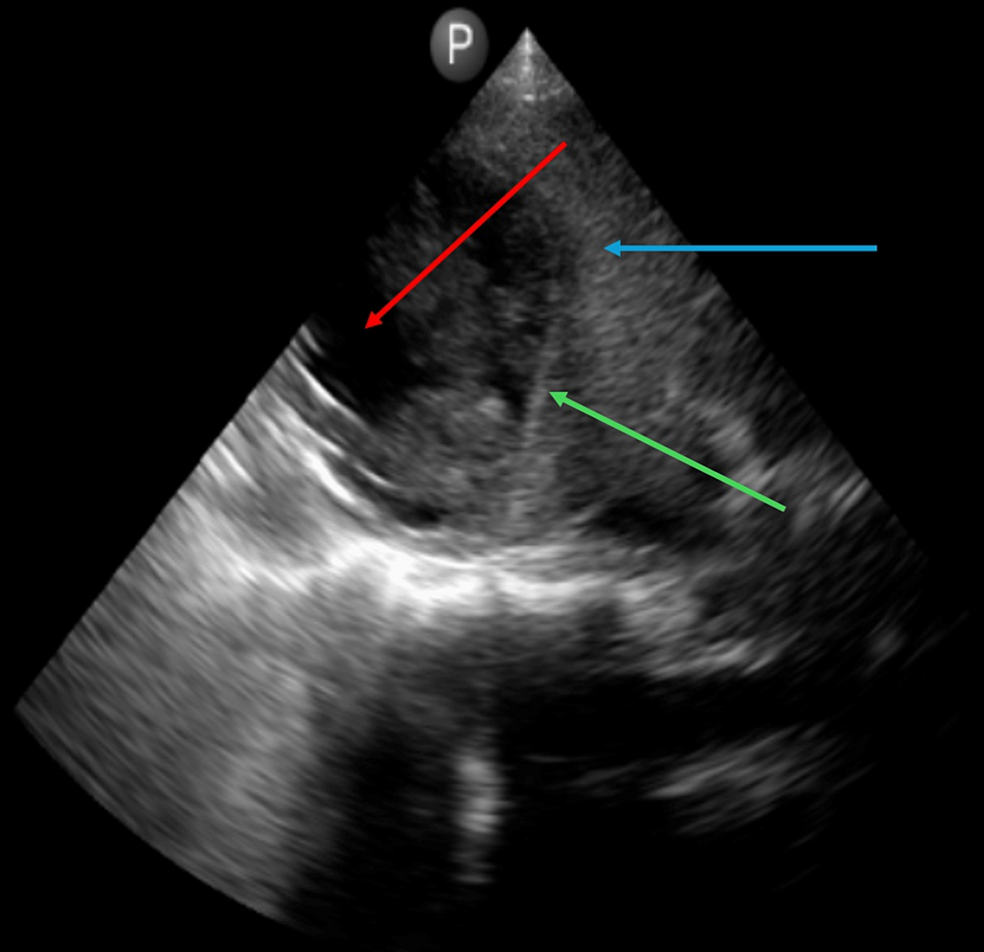
Liver POCUS image, one month after the first aspiration, demonstrating decreased abscess size. POCUS: point-of-care ultrasound. The blue arrow indicates the liver, the red arrow indicates the abscess, and the green arrow indicates the hyperechoic rim.

**Figure 4:**
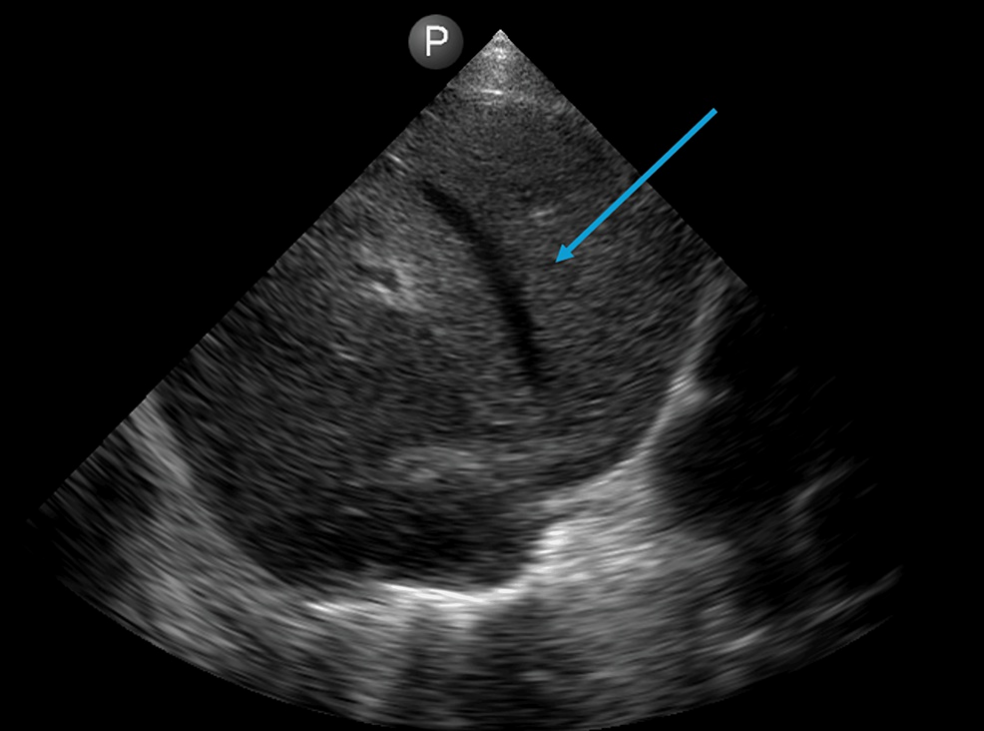
POCUS image at the time of discharge from the clinic demonstrating complete resolution of the abscess. POCUS: point-of-care ultrasound. The blue arrow indicates the liver.

On follow-up one month after discharge, the patient was feeling much improved and had no complaints. Her weight improved from 44.5 kg at presentation to 57.2 kg at discharge. The white cell count was 6.7 x 10^9^/L with 68.5% neutrophils and ESR of 24 mm/hr at the time of discharge. POCUS at this one-month follow-up visit (Figure 3) showed a decrease in the size of the abscess. However, since resolution had not been achieved, another POCUS-assisted bedside aspiration of the abscess was performed, with the removal of 160 mL of pus (for a total of 1.3 L of pus over the three bedside aspirations). There was again no bacterial growth on the culture of the aspirate. Repeat POCUS on follow-up four months after discharge from the hospital (Figure 4) demonstrated the resolution of the abscess. The patient was discharged from the clinic and has been asymptomatic for one year to date.

## Discussion

To our knowledge, this is one of the first case reports describing POCUS used in the diagnosis and percutaneous aspiration of liver abscess at the bedside by the primary medical team in a resource-limited country. POCUS as a diagnostic tool is most commonly used in critical care and emergency medicine but its use in resource-limited countries is still growing [7]. Due to limitations in diagnostic testing in our resource-constrained setting, this patient was diagnosed clinically with an amoebic liver abscess based on the appearance of the aspirate, the absence of bacterial culture growth, and sonographic improvement of the abscess after empiric antiprotozoal therapy. On US, an amoebic liver abscess can be visualized as a single hypoechoic without a capsule or rim hepatic lesion in 60% of cases, typically located in the posterior aspect of the right lobe. There may also be hyperechoic areas in the center or at the periphery [7]. The bedside POCUS done on this patient had similar features as described, further solidifying a clinical diagnosis of amoebic liver abscess. POCUS has previously been used to diagnose liver abscesses [7-9] but only one prior case report in the literature describes its use in treating this condition at the bedside without the need for radiology consultation in a resource-limited setting [6]. POCUS was used to both confirm the diagnosis of this patient and perform percutaneous aspiration of the liver abscess at the bedside in our patient.

The benefits of POCUS in resource-limited countries are increasingly recognized. Imaging modalities such as computed tomography (CT) and magnetic resonance imaging (MRI) are rarely available in low-resource settings given the cost of the machines, maintenance, staff training, and the lack of specialists needed for image interpretation. As a result, US is a key imaging modality utilized in rural and LMIC healthcare settings due to its low cost [10]. In this case, due to the non-availability of radiology services, trained personnel, and specialist services at the district level, the patient was referred to our facility for a higher level of care. These limitations can be avoided or improved by the provision of adequate clinician training on POCUS and the availability of POCUS not only in large referral hospitals but also in smaller health clinics and district hospitals [11].

Most cases of amoebic liver abscess occur in males 20-40 years of age. In this case, the patient was female but within the typical age range. Patients with liver abscess usually present with fever and right upper quadrant tenderness [1]. In studies conducted in India, an LMIC where healthcare resources are constrained [12], fever was the most common presentation and was seen in 91% of patients, followed by abdominal pain in 86% [13,14]. The patient in this case demonstrated these findings but also complained of weight loss, passing loose watery stools, loss of appetite, and vomiting. She also had jaundice. The laboratory tests of liver abscess patients may also show neutrophilic leukocytosis, raised inflammatory markers, increased alkaline phosphatase, and abnormal liver function tests [1]. These laboratory derangements were all seen in the patient in this case.

The differential diagnosis of amoebic liver abscess also includes pyogenic liver abscess, fungal liver abscess, echinococcal liver cyst, and hepatocellular carcinoma. This case was misdiagnosed as liver cancer by a US scan. The sensitivity of US for the detection of liver abscess is 85% compared with 97% for CT scan [1]. Unfortunately, CT scans may not always be accessible in a resource-limited setting. However, because of a high index of suspicion by the gastroenterologist and the use of POCUS, the diagnosis of liver abscess was established. This confirms the need to establish specialist clinics and provision of adequate clinician training on POCUS in resource-limited countries to help improve the diagnosis and treatment of liver abscesses. It is known that subsequent bacterial infection can be found in 20% of amoebic liver abscesses [11]. This is why antimicrobial guidelines in LMIC often recommend empiric therapy designed to treat both amoebic and pyogenic etiologies of liver abscess [1]. In this patient, both ciprofloxacin and metronidazole were started empirically at presentation to cover for both abscess types.

Amoebic liver abscess therapy typically includes 5-10 days of oral metronidazole or tinidazole, followed by 5-10 days of treatment with a luminal agent such as paromomycin to eliminate any remaining cysts in the gastrointestinal tract [3]. This patient was put on IV antibiotics as she was not able to tolerate oral medications, and a luminal agent was not administered because of a lack of availability in the Gambia. She subsequently experienced an improvement in her condition with a sonographic resolution of the abscess.

Based on guideline recommendations, liver abscesses less than 3 cm can be treated medically. For larger abscesses, sequential aspiration increases the likelihood of resolution [1]. In a study done in London, 60% of cases were managed with aspiration or drainage in addition to antibiotic therapy [15] and a similar study conducted in India found that nearly 70% of patients require percutaneous interventions [14]. The standard of care for drainage of liver abscesses in the Gambia is to perform a US scan and identify the area to insert the needle. The procedure is subsequently conducted blindly (without US guidance) until no further pus can be aspirated. This is usually conducted in the radiology department by a radiologist and thus requires the transport of the patient to another unit, involvement of additional resources, and delays in care. Bedside performance of the aspiration for this patient allowed for more rapid diagnosis and treatment of a highly morbid disease process. By performing this procedure at the bedside, the need to involve additional specialists and staff was also eliminated, thereby conserving resources in an already resource-limited setting.

In this case, metronidazole was started immediately, and repeated POCUS-assisted percutaneous needle aspiration was conducted on three occasions. In resource-rich settings, the standard of care for a liver abscess is drainage via interventional radiology; this is often not available in LMIC. POCUS provides the option of US-assisted percutaneous aspiration allowing for improved patient care. Potential adverse events are rare with percutaneous liver abscess aspiration, with an overall complication rate of <1% [16], but it is advisable that the care team member with the greatest POCUS proficiency conduct bedside POCUS-assisted aspiration in cases similar to the one described. This case of successful resolution of liver abscess via a combination of antibiotic therapy and serial POCUS-assisted needle aspiration demonstrates the effective use of a low-cost solution for abscess management in LMIC settings where the availability of materials and subspecialty services is limited [1].

## Conclusions

This case report describes the novel use of POCUS for the diagnosis and successful treatment of a liver abscess by the primary medical treatment team at the bedside, avoiding the need for radiology intervention and other costly resource use. In settings where radiology services are not regularly available and healthcare resources are limited, POCUS presents a potential solution that can help improve accessibility to needed interventions. With adequate training and availability of POCUS in resource-limited settings, early diagnosis and treatment of liver abscess can be achieved, thus helping to reduce its related morbidity and mortality.
